# Role of miR-144-5p in modulating lipid metabolism and potentially alleviating obesity via the PGC-1α/AMPK pathway

**DOI:** 10.3389/fvets.2025.1477593

**Published:** 2025-08-14

**Authors:** Siriguleng Yu, Chaoqun Liu, Hong Lv, Zhijun Guo, Hongqiang Yao, Xin Wen, Ying Xiu, Wa Gao, Ziyi Li, Wen Yu, Yaru Niu, Junjian Jin

**Affiliations:** ^1^College of Veterinary Medicine, Inner Mongolia Agricultural University, Huhhot, China; ^2^Key Laboratory of Clinical Diagnosis and Treatment Technology for Animal Diseases, Ministry of Agriculture, Hohhot, China; ^3^Department of Public Health, Inner Mongolia Center for Disease Control and Prevention, Hohhot, China; ^4^Inner Mongolia Academy of Agricultural & Animal Husbandry Sciences, Huhhot, China

**Keywords:** Bactrian camel, exosome, metabolism, miR-144-5p, PGC-1α

## Abstract

**Introduction:**

MiR-144-5p is differentially expressed in plasma exosomes from Bactrian camels of varying body sizes, with GO and KEGG analyses indicating that its target genes play crucial roles in lipid metabolism. PGC-1α, confirmed as a key target through RNA pull-down and dual-luciferase reporter assays, is a significant regulator of this process. This study aims to investigate the impact of miR-144-5p on lipid metabolism in a mouse model of high-fat diet (HFD)-induced obesity to elucidate the mechanistic pathways involved.

**Methods:**

Male C57BL/6 mice were assigned to groups and fed either a control diet or an HFD for 12 weeks, which was followed by a 3-week intervention with miR-144-5p. Various metabolic parameters and gene expressions were evaluated to determine the effects of this treatment.

**Results:**

The treatment significantly reduced diet-induced adiposity and decreased inflammatory responses. Additionally, it inhibited PGC-1α expression in the liver tissue of HFD-fed mice. There was an increase in CPT1 activity and fatty acid β-oxidation rate, along with the downregulation of FASN mRNA expression and the upregulation of CPT1 and ACOX1 expression, promoting fatty acid oxidation.

**Discussion:**

These findings suggest that miR-144-5p exerts anti-obesity effects by enhancing fatty acid oxidation, likely through modulation of the PGC-1α/AMPK signaling pathway. This study provides new insights into potential miRNA-based therapeutic strategies for obesity and related metabolic disorders.

## 1 Introduction

Obesity has emerged as a global epidemic, characterized by an imbalance in body fat homeostasis and a persistent state of systemic inflammation (1). Understanding the mechanisms underlying lipid metabolism is crucial in developing effective interventions for obesity. Bactrian camels, adapted to the harsh conditions of desert and semi-desert environments, exhibit remarkable biological traits such as resistance to cold, drought, and starvation. These adaptations may be closely linked to their unique lipid metabolism mechanisms, yet these mechanisms remain inadequately elucidated.

Previous research from our laboratory has demonstrated that the quantity and composition of plasma-derived exosomes in Bactrian camels vary with changes in body size. Utilizing small RNA sequencing, we have identified distinct differences in the miRNA expression profiles of plasma exosomes from camels of different body sizes (2). Particularly, miR-144-5p showed notable variability in expression levels, suggesting its potential involvement in lipid metabolism and obesity regulation.

MicroRNAs (miRNAs) are small non-coding RNAs, typically 21–25 nucleotides long, that regulate gene expression by binding to the 3′-untranslated regions (3’-UTR) of target mRNAs, thereby reducing or blocking protein synthesis (3–5). For instance, miR-21 has been shown to inhibit gluconeogenesis by downregulating phosphoenolpyruvate carboxykinase (Pepck) and glucose-6-phosphatase (G6pase) through its action on hepatic forkhead box O1 (Foxo1) (6). This highlights the critical role of miRNAs as post-transcriptional regulators in a range of metabolic disorders, including obesity. We hypothesize that exosomal miR-144-5p is a significant regulator of lipid metabolism in Bactrian camels.

Peroxisome proliferator-activated receptor-gamma coactivator-1 alpha (PGC-1α) is a transcriptional coactivator instrumental in the regulation of mitochondrial biogenesis, lipid metabolism, and energy homeostasis (7). *In vitro* and *in vivo* studies have shown that PGC-1α enhances hepatic fatty acid oxidation and reduces triglyceride storage and secretion (8). Nevertheless, recent studies indicate that miRNAs can inhibit PGC-1α expression, highlighting a potential therapeutic target for obesity through miRNA intervention (9, 10). Using bioinformatics tools, we identified several potential target sequences of miR-144-5p among over 100 mRNAs involved in lipid metabolism and energy regulation. We propose that miR-144-5p may influence obesity progression by interacting with PGC-1α. However, the detailed role of this interaction remains to be elucidated in the context of obesity.

Based on the above findings, the present study aims to investigates the effects of miR-144-5p administration on lipid metabolism in high-fat diet (HFD)-induced obese mice model, with a specific focus on the miR-144-5p/PGC-1α axis. This research will examine the regulatory role of miR-144-5p in modulating key metabolic pathways and genes associated with obesity and lipid metabolism. By analyzing the interactions between miR-144-5p and its target genes, this study seeks to elucidate the underlying mechanisms by which miR-144-5p influences metabolic dysfunctions in obesity, thereby contributing to the development of potential therapeutic strategies for obesity management.

## 2 Materials and methods

### 2.1 Isolation and characterization of plasma-derived exosomes

Exosomes were isolated from plasma samples of Bactrian camels (group A and B, n = 5 for each group, thin and normal camels respectively) at 4°C, following a modified protocol based on previously described methods (11). In brief, blood samples were diluted with isopycnic PBS and centrifuged at 300 g for 10 min. The supernatant was then collected into a new tube and subjected to sequential centrifugation at 2000 g for 10 min, 12,000 g for 30 min, and 120,000 g for 70 min. The sediments were resuspended in pre-cooled PBS (2,500 mL) to obtain exosomes and store at −80°C. Exosome concentrations were quantified using a BCA protein assay kit (Boster Biological Technology Co., Ltd., Wuhan, China) according to the manufacturer’s guidelines. For further analysis, 50 mL of the isolated solution was mixed with 10 mL of PBS and centrifuged again at 120000 g for 70 min at 4°C. The sediments from this step were resuspended in 50 mL of PBS and used for transmission electron microscopy (TEM) and Nano particle tracking analyzer (NTA). TEM (Tecnai G2 spititi, FEI company, OR, United States) was utilized to examine the morphology and ultrastructure of the isolated exosomes, following methods established in prior studies (12). Additionally, exosomes size distribution was assessed using a ZetaView^®^ NTA (ZetaView Particle Metrix, Germany) in accordance with the protocol described by Soares Matins et al. (13). The research protocols were approved by the Experimental Animal Welfare and Ethics Committee of Inner Mongolia Agricultural University (No. NND2023059) on March 6, 2023.

### 2.2 Verification of miR-144-5p expression levels by quantitative real-time PCR (qRT-PCR)

Total RNA was extracted from exosomes using the exosomal total RNA extraction kit (77,023, QIAGEN, Shanghai, China) following the manufacturer’s protocol. Total intracellular RNA was extracted using Trizol (15,596,026, Invitrogen, Beijing, China), which was detected by an ABI 7500 RT-qPCR instrument (Thermo Fisher Scientific, Waltham, MA, United States). Primers were designed and synthesized by Sangon Biotech (Shanghai, China; Supplementary Table 1), with U6 as an internal control for miR-144-5p. All results were normalized based on the 2^−ΔΔCt^ method.

### 2.3 Prediction and selection of miR-144-5p target genes

Using databases of miRBase,^1^ BLAST,^2^ and CLUSTALW,^3^ the mature sequences of miR-144-5p were compared across different species to evaluate their conservation. Then, target genes of miR-144-5p were predicted using TargetScan^4^ and miRWalk,^5^ determining binding sites and selecting the intersection results from the two systems as the focus for further analysis.

### 2.4 GO and KEGG enrichment analysis of miR-144-5p target genes

The target genes were subjected to Gene Ontology (GO) enrichment analysis using DAVID 6.8^6^ and Kyoto Encyclopedia of Genes and Genomes (KEGG) pathway annotation analysis.^7^ KEGG enrichment analysis was performed by relevant bioinformatics software, and visualization was completed in R Studio 4.2.1 and on the bioinformatics online plotting website.^8^

### 2.5 Cell culture

Human embryonic kidney 293 T cells (Stem Cell Bank, Chinese Academy of Sciences, Shanghai) were maintained in Dulbecco’s modified Eagle’s medium (DMEM; SH30022.01, HyClone, Shanghai) supplemented with 10% fetal bovine serum (FBS; SH30070.03, HyClone, Shanghai), along with 100 units/mL penicillin and 100 μg/mL streptomycin (15140–122, Invitrogen, Shanghai). The cultures were kept at 37°C in an atmosphere of 5% CO_2._

### 2.6 Biotinylated miRNA pull down assay for identifying miRNA targets

To investigate direct miRNA-mRNA interactions using the miRNA pull down assay, start by synthesizing biotin-labeled miR-144-5p and a scrambled control miRNA. Seed 293 T cells 1 day before transfection in 10 cm tissue culture dish in duplicate. 24 h later, transfect the cells with either the control miRNA or the 3’biotin-labeled miRNA (Bio-NC group and Bio-miR-144-5p group) at a final concentration ranging from 10 to 100 nM. After a 48-h post-transfection period, wash the cells with ice-cold PBS and collect whole cell lysates. Lyse the cells using a lysis buffer, followed by centrifugation to clarify the lysates. Meanwhile, prepare and wash Streptavidin-Dynabeads, then incubate them with the cell lysates and yeast tRNA to perform an overnight pull-down on a rotator at 4°C. Following this, isolate RNA using Trizol LS and precipitate it with ethanol, resuspending the purified RNA in nuclease-free water. Set up RT-PCR reactions for mRNA using biotin-labeled RNA and input samples, along with reverse transcription for miRNA. Finally, conduct quantitative PCR (qPCR) in triplicate to assess target mRNAs and miRNAs, using U6 as a normalization control.

### 2.7 Dual-luciferase reporter assay

For the luciferase assays, plasmids containing the wild-type (WT) and mutant-type (MUT) 3’-UTR of PGC-1α gene were constructed within the pMIR-REPORT luciferase vector (E1910, Promega, Beijing). 293 T cells were cultured in 12-well plates until they reached 60 to 70% confluence. Cells were then divided into four groups: NC + WT (mimic NC + PGC-1α WT), miR + WT (miR-144-5p + PGC-1α WT), NC + MUT (mimic NC + PGC-1α MUT), and miR + MUT (miR-144-5p + PGC-1α MUT). Co-transfections were performed using Lipofectamine 2000 (11,668,019, Invitrogen, Shanghai), with each group receiving the appropriate combination of miRNA and luciferase reporter plasmids. To normalize transfection efficiency, a Renilla luciferase vector was also included in each transfection. Following a 5-h incubation in serum-free DMEM with the transfection mixture, the medium was replaced with DMEM containing 10% FBS, and cells were incubated at 37°C with 5% CO_2_. Cell lysates were collected at 24 and 48 h post-transfection for analysis. Luciferase activity was measured using the Dual-Luciferase Reporter Assay System (E2920, Promega, Beijing), and the Firefly luciferase activity was normalized to that of Renilla luciferase to determine the relative luciferase activity.

### 2.8 Animals and designs

Forty male C57BL/6 J mice (HFK Biotechnology Co., Ltd., Beijing, China) were maintained in the animal room under specific pathogenfree conditions (22 ± 2°C, 40–70% relative humidity, 12 h light–dark cycle). After a one-week acclimatization, all mice were randomly divided into a control group (Ctrl, *n* = 10, fed a normal basal diet) and an experimenal group (Exp, *n* = 30, fed a HFD). During 12 weeks of feeding to induce obesity, the body weight and length of all mice were measured weekly to calculate the Lee’s index.

### 2.9 Intravenous administration of miR-144-5p agomir and antagomir

The obese mice were randomly assigned into three groups: model group (Mod), agomir group (Ago), and antagomir group (Ant; *n* = 10 for each group). miR-144-5p agomir (500 nmol/kg), and miR-144-5p antagomir (500 nmol/kg) were administered via tail vein injection once every 3 days for 3 weeks. For comparison, the model group of obese mice were received equal amounts of PBS treatment, as a negative control. All agomirs and antagomirs were synthesized by Shanghai GenePharma Co., Ltd. (Shanghai, China) and prepared following the manufacturer’s instructions.

### 2.10 Serum biochemical index analysis

The levels of serum total cholesterol (TC), triglyceride (TG), high-density lipoprotein cholesterol (HDL-C), and low-density lipoprotein cholesterol (LDL-C) were measured with the blood lipid assay kits (Nanjing Jiancheng Bioengineering Institute, Nanjing, China).

### 2.11 Inflammatory cytokine detection

The levels of key inflammatory cytokines, specifically inducible nitric oxide synthase (iNOS), tumor necrosis factor alpha (TNF-*α*), arginase 1 (Arg-1), and interleukin 10 (IL-10) in serum and liver tissue, were evaluated using ELISA kits (Sigma, Beijing, China). The assays were performed according to the manufacturer’s detailed instructions.

### 2.12 CPT1 activity assay

Carnitine palmitoyltransferase-1 (CPT1) activity assay was performed as previously described (14). Briefly, mice liver whole protein samples (30 μg) were mixed with 5, 5′-dithio-bis(2-nitrobenzoic acid; DTNB, D8130, Sigma, Beijing, China) at a final concentration of 1 mM in a reaction buffer (20 mM Tris, pH 8.0, 1 mM EDTA) and incubated at room temperature for 30 min. To initiate the reaction, solutions of palmitoyl-CoA (P9716, Sigma, Beijing, China, at a final concentration of 100 μM) and L-carnitine (8.40092, Sigma, Beijing, China, at a final concentration of 5 mM) were added to the reaction mixture. A negative control was set up using the reaction buffer instead of palmitoyl-CoA. Absorbance was measured at 412 nm at 1 min intervals for 90 min. The enzyme activity was calculated as nmol of CoA-SH released/min/mg protein.

### 2.13 Fatty acid *β*-oxidation rate detection

Detection of fatty acid β-oxidation rate was conducted in accordance with previous descriptions (15). Mice liver tissue was homogenized in PBS buffer, followed by the isolation of mitochondria using a Cell Mitochondrial Isolation Kit (C3601, Beyotime, Shanghai, China). After isolating the mitochondria, they are treated with 0.25 mM palmitic acid and varying concentrations of mangiferin (12.5, 25, and 50 μM) for 24 h. The fatty acid β-oxidation rate is then measured according to the fatty acid β-oxidation Kit (BR00001, Assay Genie, Whhan, China) by determining the reduction rate of ferricyanide dependent on the oxidation of palmitoyl carnitine.

### 2.14 Quantitative real-time PCR

The total RNA content of the samples was extracted using TRIzol reagent (15,596,018, Invitrogen, Beijing, China). Then, 1 μg of total RNA was reverse-transcribed into cDNA with a commercial cDNA Synthesis Kit (WE0132, Beijing Baiao Leibo Technology Co., Ltd., Beijing, China). A fluorescent qRT-PCR instrument (Applied Biosystems, United States) and 2 × Fast HS SYBR qPCR Mixture kit (D7260, Beyotime, Shanghai, China) were used for fluorescence quantification. The relative expression of all genes was calculated according to the 2^−ΔΔCt^ method and normalized using GAPDH as the housekeeping gene. Primer sequences are shown in Supplementary Table 1.

### 2.15 Western blot analysis

Total protein was extracted with lysis buffer and quantified using the BCA Protein Assay Kit (YT036, Beijing Baiao Leibo Technology Co., Ltd., Beijing, China). Equal amounts of protein were separated by SDS-PAGE and subsequently transferred to polyvinylidene fluoride membranes. The membranes were blocked with 5% skim milk powder solution and incubated overnight (4°C) with primary antibodies against *β*-actin (1,10,000), Arg-1 (1:1,000), TNF-*α* (1,1,000), and PGC-1α (1,5,000, MBS840561, MyBioSource, Wuhan, China). After washing with TBST 3 times, the secondary antibodies (1,5,000, A0208, Beyotime, Shanghai, China) were incubated for 2 h at room temperature, followed by another 3 washes with TBST. Finally, protein signals were detected and scanned using the Quantitative Fluorescence Imaging Systems (Beijing SinSage Technology Co., Ltd., Beijing, China).

### 2.16 Histopathology

The collected liver tissues were fixed in 4% paraformaldehyde solution (v/v, 0.1 mol/L PBS, pH 7.4) for 24 h. The tissues were embedded in paraffin and cut into sections of 5 μm thickness, and the sections were stained using Hematoxylin and Eosin (H&E). The tissue structures were then observed using a microscope (Olympus Corporation, Beijing, China).

### 2.17 Data analysis

All data were analyzed using SPSS version 23.0, employing one-way analysis of variance (ANOVA) to assess differences among groups, followed by Tukey’s HSD *post-hoc* test if a significant difference (*p* < 0.05) was detected. For comparisons between two independent groups, Student’s t-test was applied. Image processing was conducted with ImageJ software, and graphical representations of the data were generated using GraphPad Prism version 9.0. All statistical tests were conducted at a significance level of *p* < 0.05, and results were expressed as mean ± standard deviation (SD).

## 3 Results

### 3.1 Morphological identification and nanoparticle size analysis of exosomes

**Figure 1:**
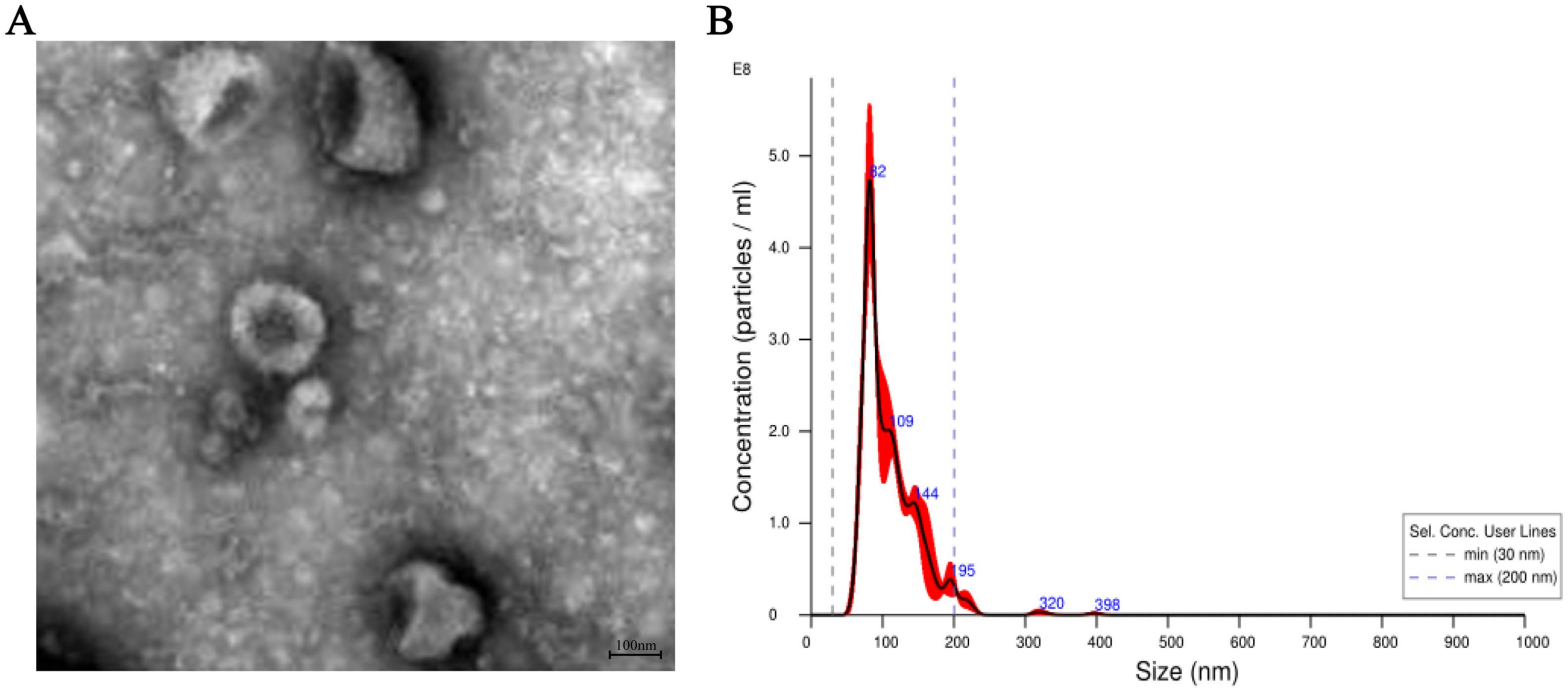
Characterization of exosomes from the plasma of Bactrian camels. **(A)** The morphology of exosomes from the plasma of Bactrian camels visualized using a transmission electron microscopy. Scale bar = 100 nm. **(B)** The exosomes size distributions measured by nanoparticle tracking analysis.

Exosomes were isolated from the plasma of two group camels using ultracentrifugation, followed by observation under transmission electron microscopy. The results showed that exosomes exhibited cup-shaped or round morphologies, with intact structures of varying sizes, approximately 100 nm in diameter (Figure 1A). Nano-particle tracking analysis revealed that the main peak particle sizes of exosomes from the plasma were approximately 110.1 nm, with an overall size distribution ranging from 30 nm to 200 nm (Figure 1B). These results are consistent with previous reports on the morphological characteristics and nanoparticle sizes of exosomes (16).

### 3.2 Expression level of miR-144-5p in exosomes from different groups

**Figure 2:**
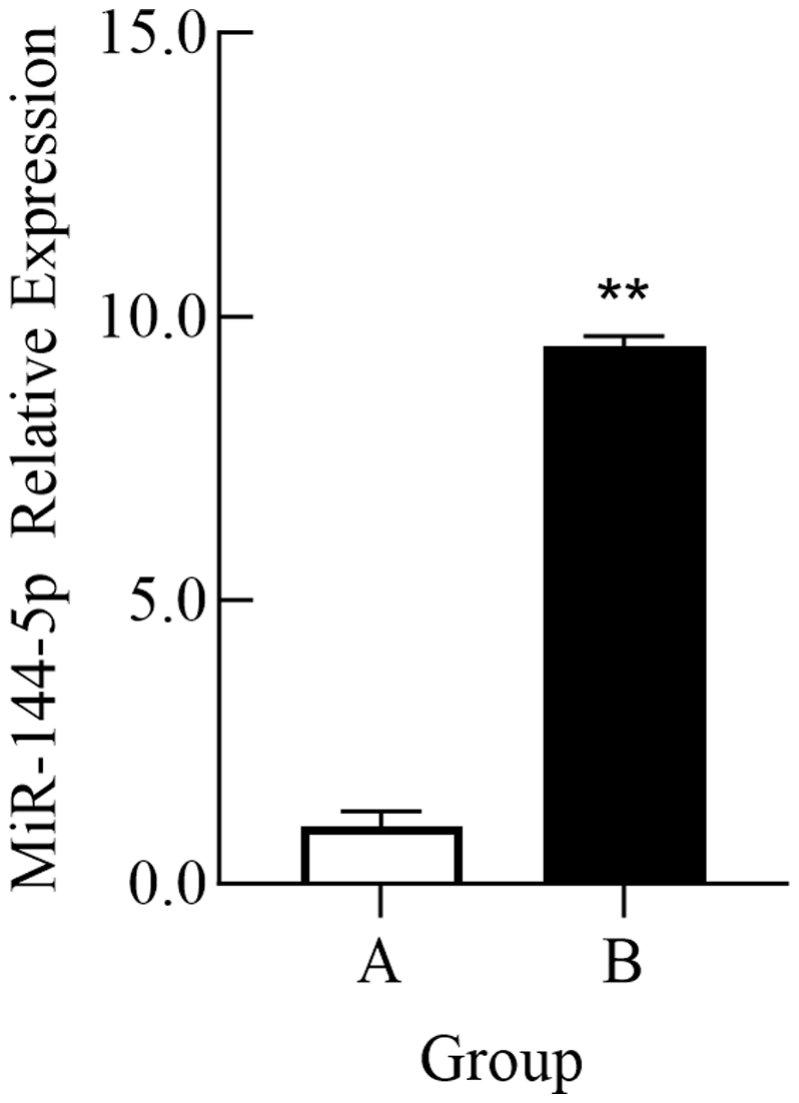
Comparison of relative expression of miR-144-5p in plasma exosomes of Bactrian camel in group A and group B. **(A)** Group A; **(B)** Group B. **indicates an extremely significant difference compared to the control group (*p* < 0.01).

For validating the sequencing results, exosomal RNA samples from groups A and B were analyzed by qRT-PCR. As shown in Figure 2, the expression level of miR-144-5p in exosomes from group A was significantly higher than that in group B (*p* < 0.01), which is consistent with the sequencing results.

### 3.3 Analysis of sequence conservation and GO and KEGG enrichment of target genes

The evolutionary conservation of miR-144-5p was analyzed using BLAST and CLUSTALW, revealing high sequence similarity across humans (*Homo sapiens*), mice (*Mus musculus*), rats (*Rattus norvegicus*), cows (*Bos taurus*), and chickens (*Gallus gallus*). Target prediction using TargetScan and miRWalk identified a putative binding site for miR-144-5p in the 3′-UTR of PGC-1α, with a 7-nucleotide seed match (positions 2–8 of miR-144-5p).

**Figure 3:**
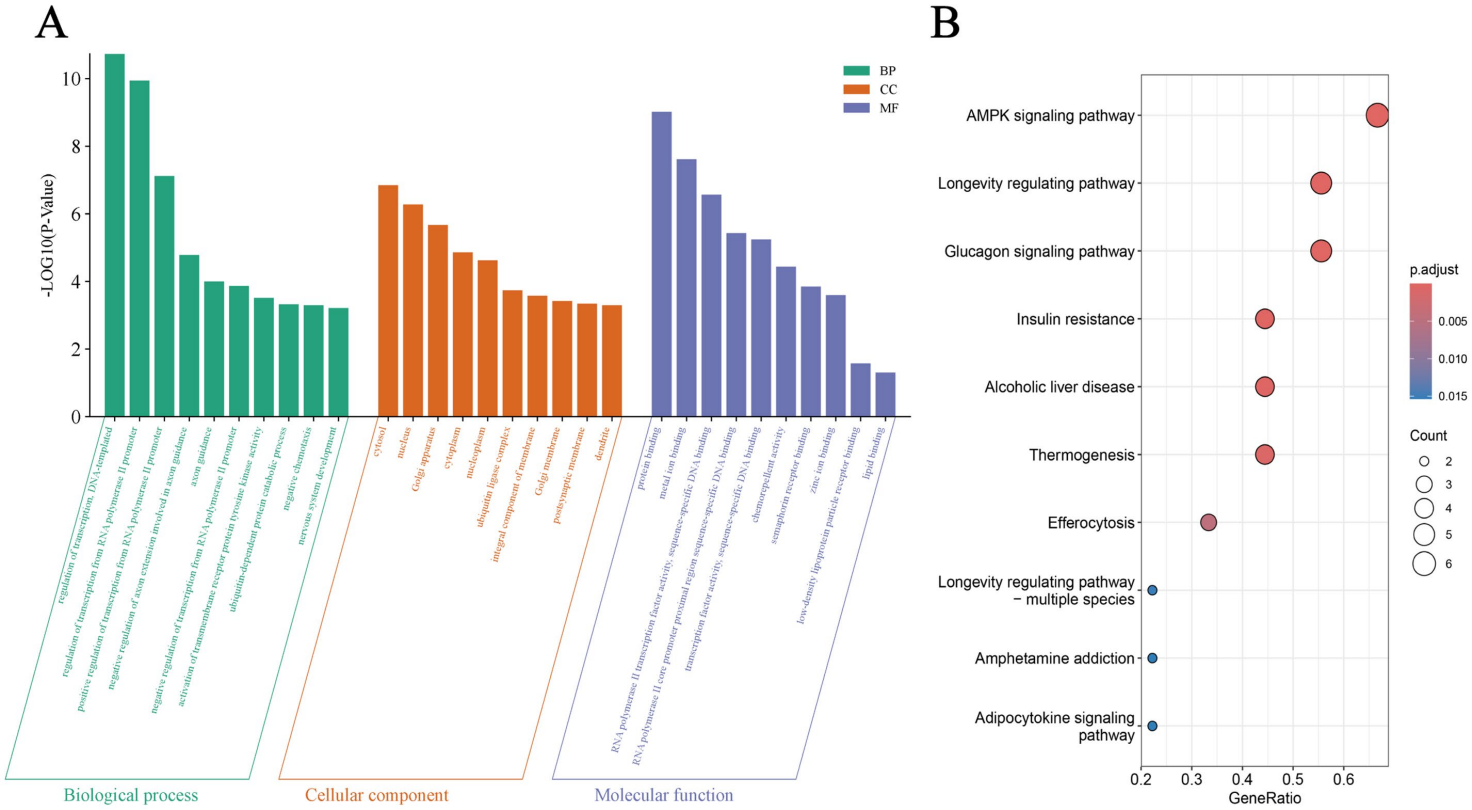
GO and KEGG enrichment of target genes. **(A)** Gene Ontology (GO) terms of miR-144-5p target genes in biological process, cellular component, and molecular function. **(B)** Kyoto Encyclopedia of Genes and Genomes (KEGG) pathways enrichment of miR-144-5p target genes.

The overlapping results from TargetScan and miRWalk predictions of miR-144-5p target genes were subjected to GO and KEGG enrichment analysis. According to the GO enrichment analysis, they were significantly related to “regulation of transcription, DNA-templated,”“regulation of transcription from RNA polymerase II promoter” and “positive regulation of transcription from RNA polymerase II promoter” in biological process (BP) GO terms, and “cytosol,” “nucleus,” and “Golgi apparatus” in cellular component (CC) GO terms, as well as “protein binding,” “metal ion binding” and “lipid binding” in molecular function (MF) GO terms (Figure 3A). Additionally, the significantly enriched KEGG pathways included “AMPK signaling pathway,” “Longevity regulating pathway,” “Glucagon signaling pathway,” and “Insulin resistance” (Figure 3B). Notably, the enrichment of “lipid binding” (MF) and “AMPK signaling pathway” (KEGG) underscores the potential role of miR-144-5p in lipid metabolism.

### 3.4 Interaction between miR-144-5p and PGC-1α

**Figure 4:**
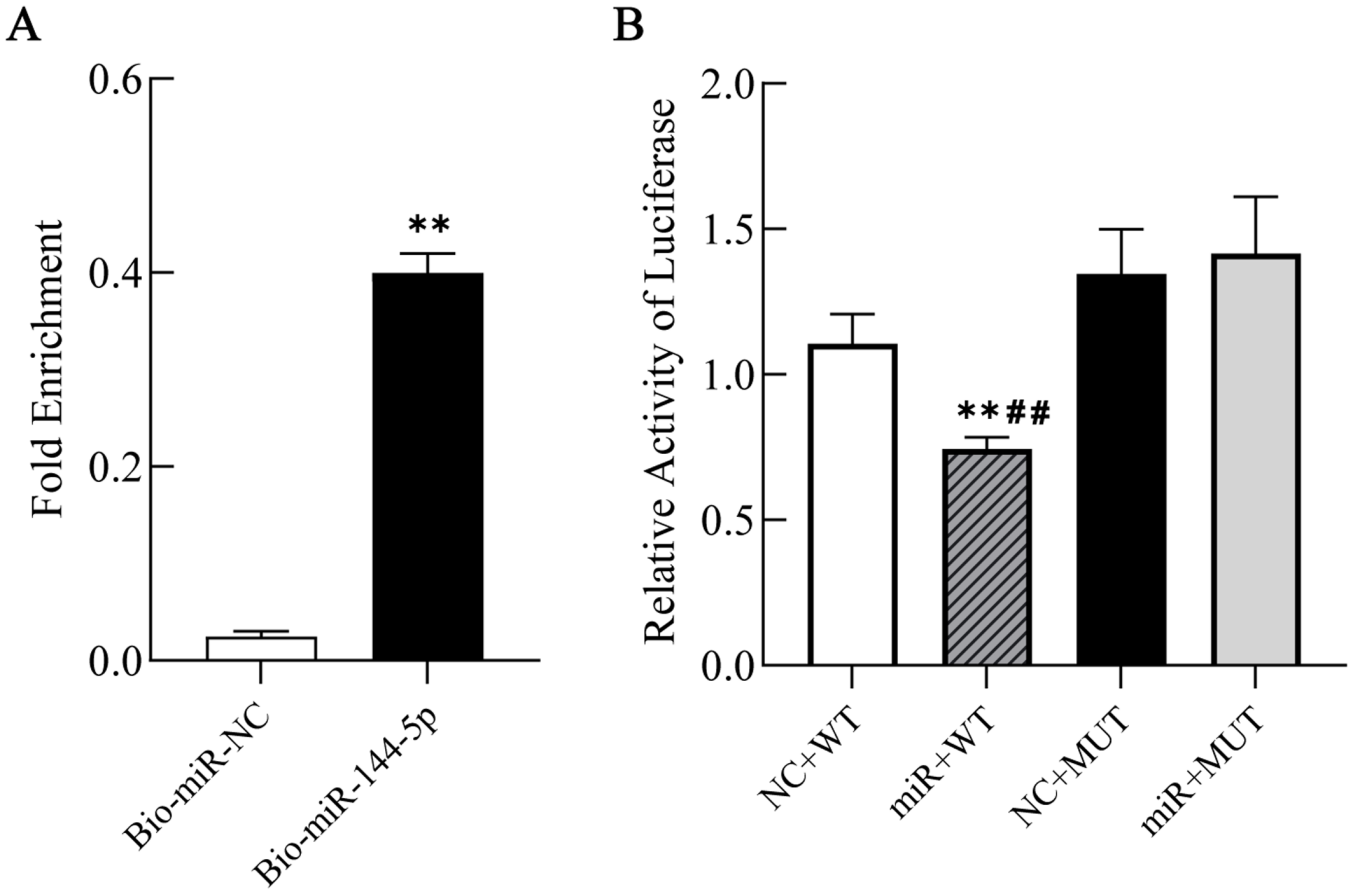
Interaction between miR-144-5p and PGC-1*α*. **(A)** Enrichment of PGC-1α mRNA binding to miR-144-5p by RNA pull-down assay followed by qRT-PCR detection. **(B)** Binding of miR-144-5p to PGC-1α 3’-UTR by luciferase reporter assay. NC + WT: PGC-1α wild type sequences with miR-144-5p-NC; miR + WT: PGC-1α wild type sequences with miR-144-5p-mimics; NC + MUT: PGC-1α mutant sequences with miR-144-5p-NC; miR + MUT: PGC-1α mutant sequences with miR-144-5p- mimics.

RNA pull-down assay was conducted to enrich RNA binding to miR-144-5p, followed by qRT-PCR detection to clarify the binding status between miR-144-5p and the target gene PGC-1α. The experimental results showed a significant enrichment of PGC-1α mRNA in the Bio-miR-144-5p group compared to the Bio-NC group (*p* < 0.01), indicating that PGC-1α mRNA interacted with miR-144-5p (Figure 4A).

To confirm the direct binding of miR-144-5p to the predicted seed sequence within the PGC-1α 3’-UTR, we constructed luciferase reporter plasmids of the PGC-1α 3′-UTR containing either the wild-type or mutant forms of the potential miR-144-5p targeting sites. Luciferase reporter assay revealed that miR-144-5p significantly suppressed the luciferase activity of the wild-type reporter (*p* < 0.01), whereas no inhibition was observed for the mutant construct (Figure 4B). These results confirmed that miR-144-5p could directly regulate PGC-1α.

### 3.5 MiR-144-5p attenuates obesity and hepatic steatosis

**Figure 5:**
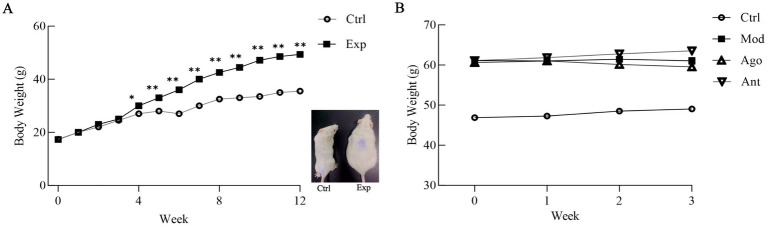
Body weight changes of obese mice. **(A)** After 12 weeks of feeding with a HFD, the final body weight of HFD-fed mice (Exp) was significantly higher than that of the normal basal diet-fed mice (Ctrl; 42.85%, *p* < 0.01). Values are means ± SD (*n* = 10 mice each). *p < 0.05, **p < 0.01, relative to control group. Ctrl: control group; Exp: experiment group. A representative picture of a Ctrl and a Exp mouse after 12-weeks on HFD (right panel). **(B)** Comparison of body weight gain between Ctrl, Mod, Ago, Ant mice on 15-weeks on HFD. Mod, model group; Ago, agomir group; Ant, antagomir group.

After 12 weeks of feeding with a HFD, the final body weight of HFD-fed mice (Exp) was significantly higher than that of the Ctrl group (42.85%, *p* < 0.01; Figure 5A). However, this increase was mitigated by the intervention of miR-144-5p, which demonstrated a trend toward weight reduction in the HFD mice (Figure 5B). These results suggest that miR-144-5p treatments reduces body weight in diet-induced obese mice.

**Figure 6:**
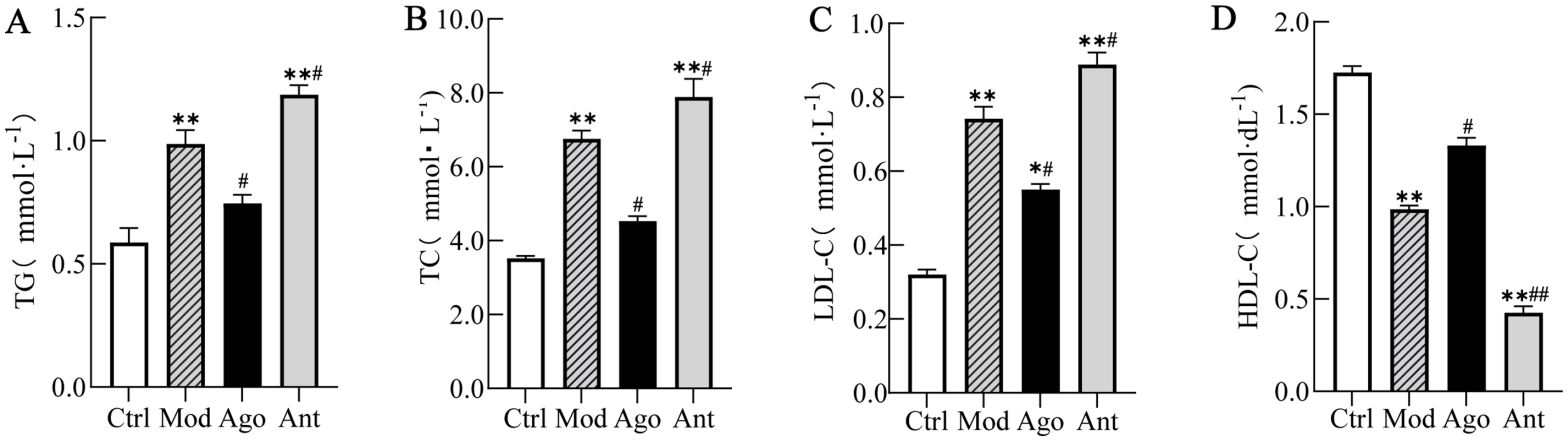
Serum lipid profile. Serum lipid profiling revealed that HFD feeding significantly increased TG, TC, and LDL-C, while decreasing HDL-C compared to the Ctrl group (*p* < 0.01). Notably, miR-144-5p intervention significantly reversed these lipid abnormalities in the obese mice. **(A)** Serum triglyceride (TG) levels. **(B)** Serum total cholesterol (TC) levels. **(C)** Serum low-density lipoprotein cholesterol (LDL-C) levels. **(D)** Serum high-density lipoprotein cholesterol (HDL-C) levels. Data shown are mean ± SD. *p < 0.05, **p < 0.01, relative to control group, #*p* < 0.05, ##*p* < 0.01, relative to model group.

Serum lipid profiling revealed that HFD feeding significantly increased TG, TC, and LDL-C, while decreasing HDL-C compared to the Ctrl group (*p* < 0.01). Notably, miR-144-5p intervention significantly reversed these lipid abnormalities in the obese mice (Figure 6).

**Figure 7:**
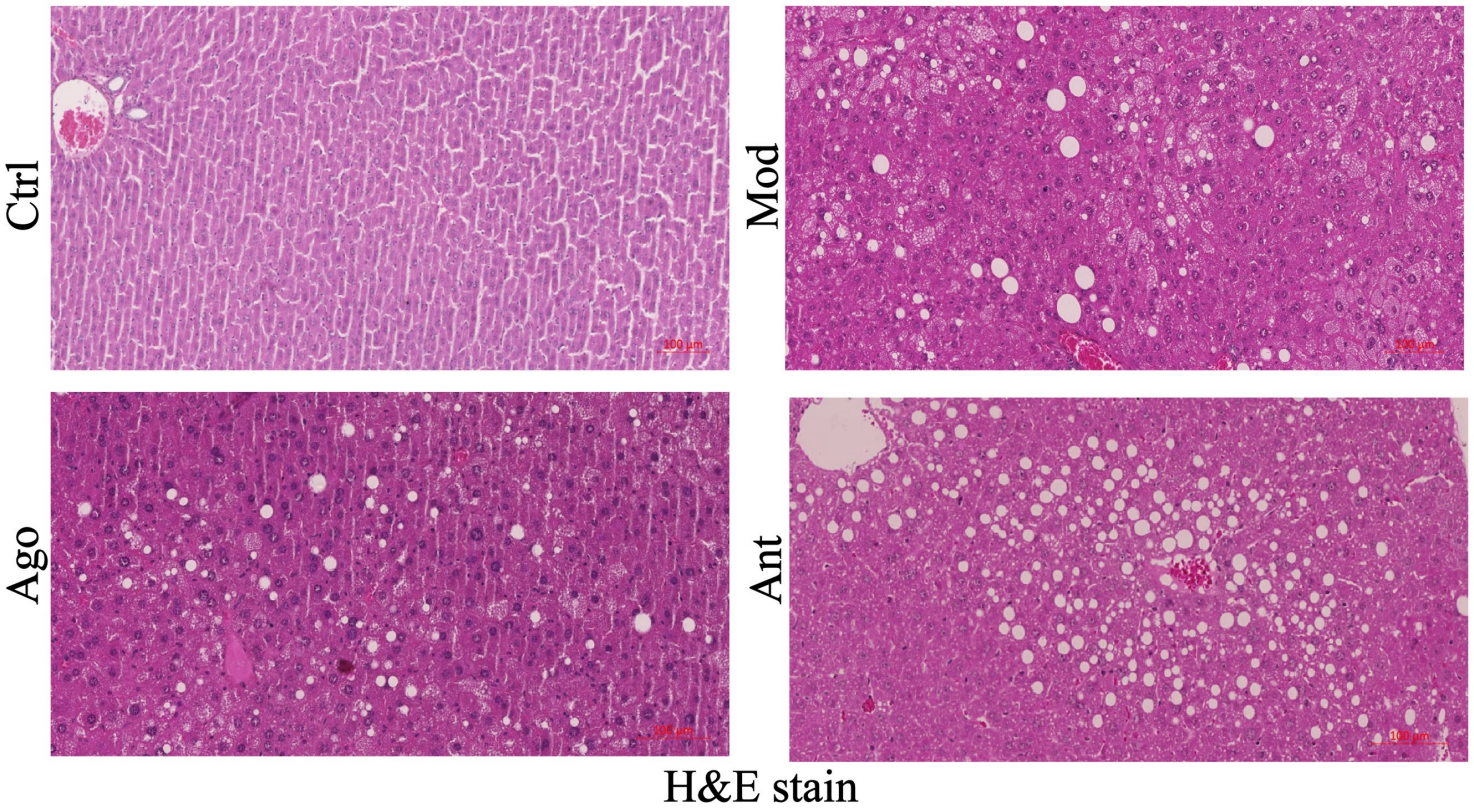
Representative H & E stain of liver tissue of mice, scale bar: 100 μm. miR-144-5p intervention mice (Ago) are protected from HFD-induced hepatic steatosis.

Histopathological analysis of liver sections (H&E staining) demonstrated severe hepatic steatosis in HFD-fed obese mice, characterized by macrovesicular steatosis, hepatocyte ballooning, and inflammatory cell infiltration. In contrast, miR-144-5p agomir treatment reduced lipid accumulation and ameliorated hepatocellular injury (Figure 7). Collectively, these findings indicate that miR-144-5p attenuates diet-induced obesity and exerts hepatoprotective effects in obese mice.

### 3.6 MiR-144-5p regulates PGC-1*α* expression and attenuates inflammatory responses

**Figure 8:**
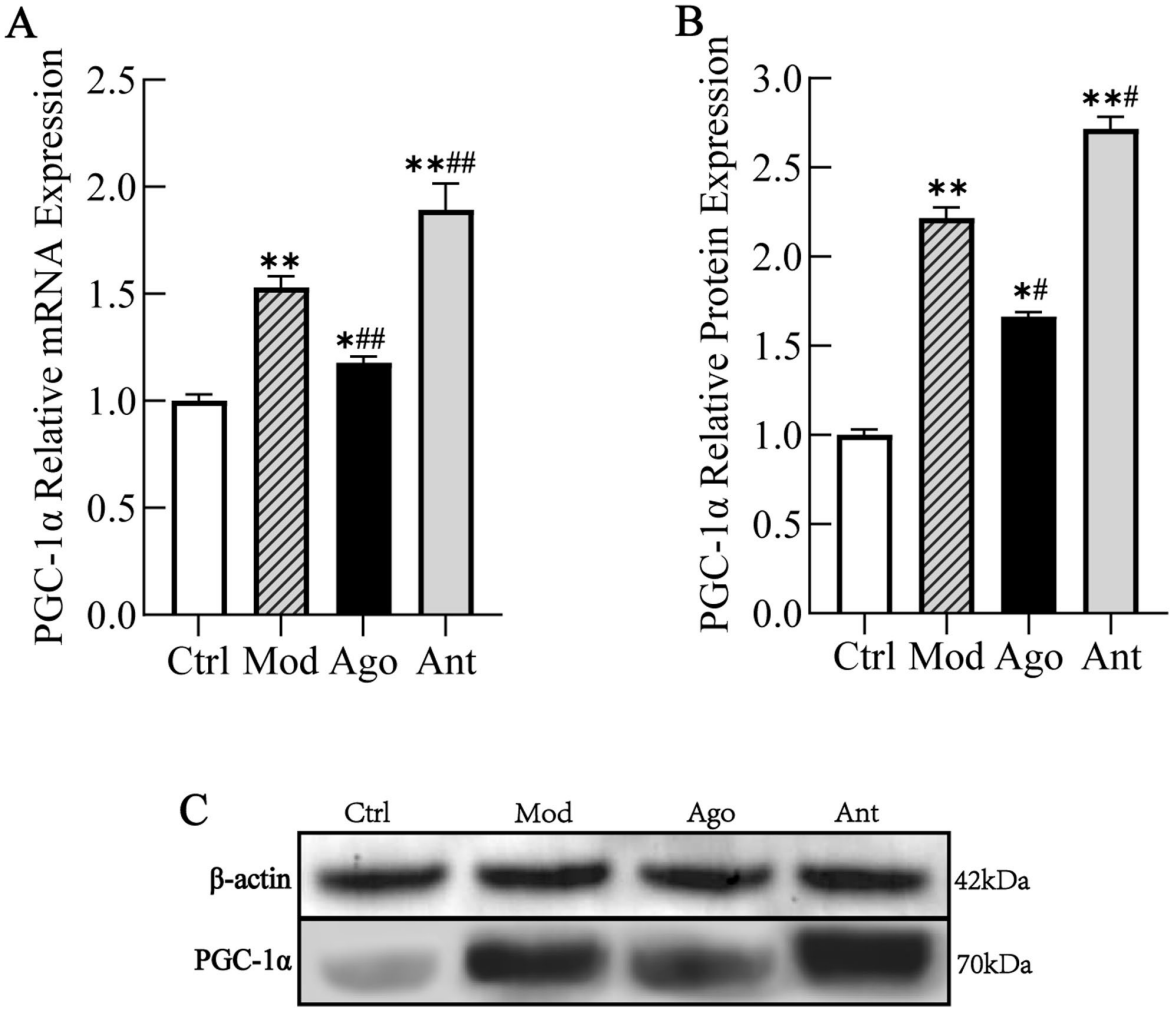
MiR-144-5p Regulates PGC-1α Expression. The expression level of PGC-1α was significantly upregulated in HFD-fed mice compared to the Ctrl group (*p* < 0.01), which was significantly reversed by the intervention of miR-144-5p. **(A)** mRNA expression level of PGC-1α. **(B)** Protein expression level of PGC-1α. **(C)** WB analysis. Data shown are mean ± SD. *p < 0.05, **p < 0.01, relative to control group, #*p* < 0.05, ##*p* < 0.01, relative to model group.

**Figure 9:**
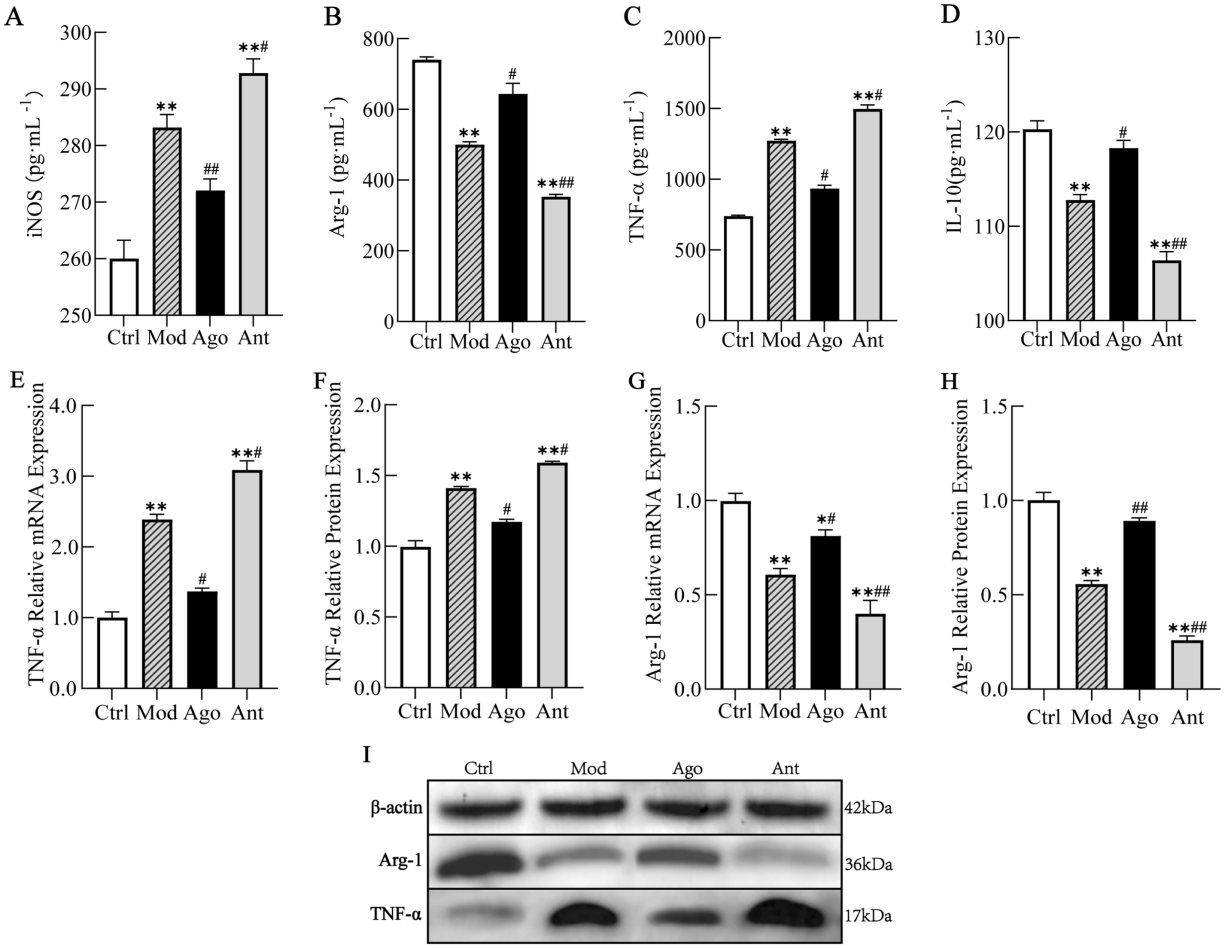
MiR-144-5p attenuates inflammatory responses. Consistent with serum analyses, HFD feeding induced a pro-inflammatory state characterized by elevated TNF-α and iNOS (*p* < 0.01), and suppressed Arg-1 and IL-10 expression (*p* < 0.01) in liver tissue compared to the Ctrl group. These changes were significantly reversed by miR-144-5p agomir administration (*p* < 0.05). **(A)** Serum level of iNOS. **(B)** Serum level of Arg-1. **(C)** Serum level of TNF-α. **(D)** Serum level of IL-10. **(E)** mRNA expression level of TNF-α. **(F)** Protein expression level of TNF-α. **(G)** mRNA expression level of Arg-1. **(H)** Protein expression level of Arg-1. **(I)** WB analysis. Data shown are mean ± SD. *p < 0.05, **p < 0.01, relative to control group, #*p* < 0.05, ##*p* < 0.01, relative to model group.

The expression levels of PGC-1α and inflammatory markers were evaluated in HFD-fed mice. As shown in Figure 8, the expression level of PGC-1α was significantly upregulated in HFD-fed mice compared to the Ctrl group (*p* < 0.01), which was significantly reversed by the intervention of miR-144-5p. Consistent with serum analyses, HFD feeding induced a pro-inflammatory state characterized by elevated TNF-α and iNOS (*p* < 0.01), and suppressed Arg-1 and IL-10 expression (*p* < 0.01) in liver tissue compared to the Ctrl group. These changes were significantly reversed by miR-144-5p agomir administration (*p* < 0.05; Figure 9). Combined with the target validation data, these results indicate that miR-144-5p suppresses PGC-1α expression and ameliorates obesity-associated inflammation in diet-induced obese mice.

### 3.7 MiR-144-5p enhances fatty acid oxidation by regulating key enzymes

To elucidate the mechanism underlying the anti-obesity and hepatoprotective effects of miR-144-5p, we analyzed hepatic CPT1 activity and fatty acid oxidation (FAO) rates in HFD-fed mice. Additionally, the mRNA expression levels of key enzymes involved in fatty acid metabolism, including CPT1, fatty acid synthase (FASN), and acyl-CoA oxidase 1 (ACOX1) were evaluated by qRT-PCR.

**Figure 10:**
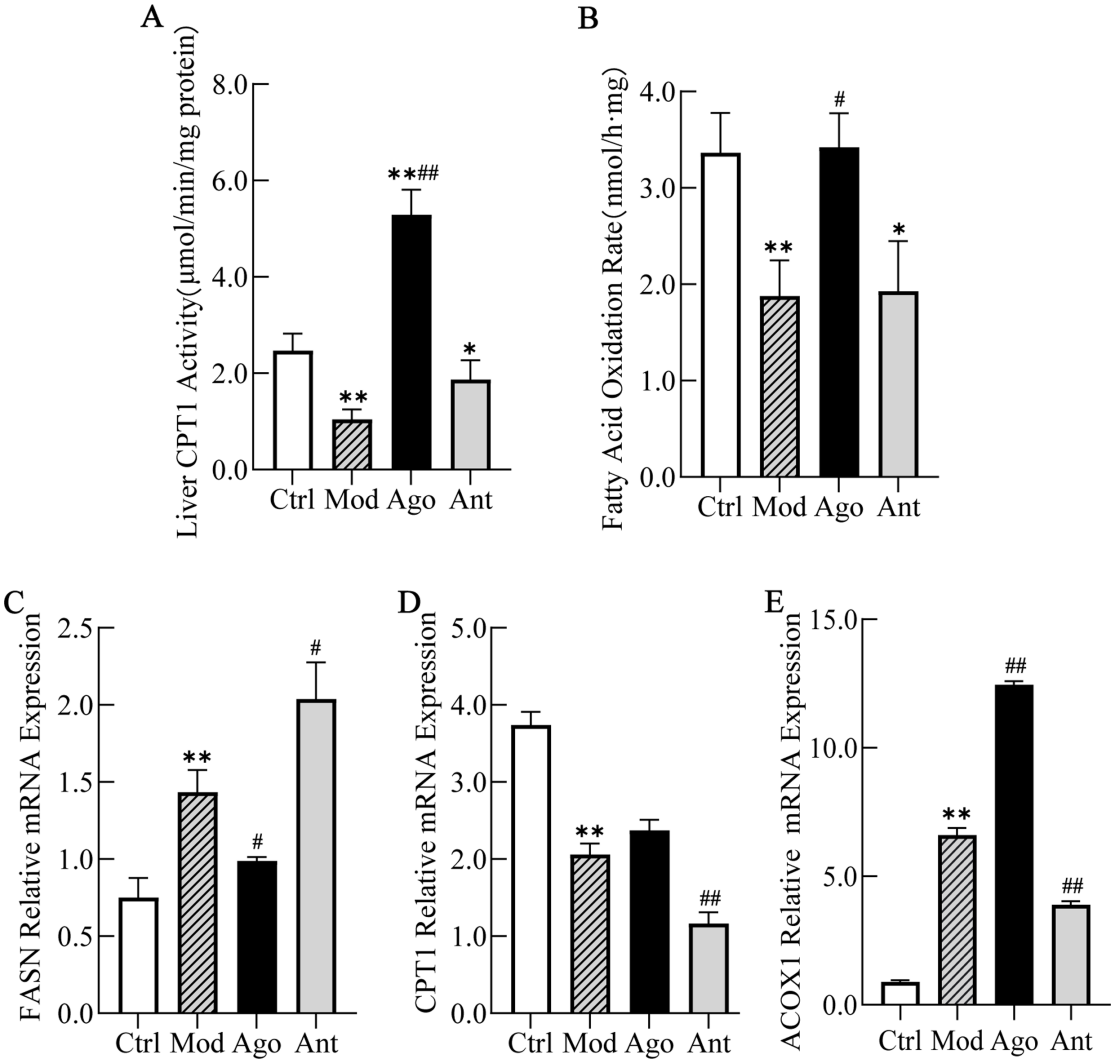
MiR-144-5p enhances fatty acid oxidation by regulating key enzymes. **(A)** MiR-144-5p intervention significantly enhanced CPT1 activity (*p* < 0.01). **(B)** MiR-144-5p intervention significantly increased the hepatic FAO rate in the liver of obese mice (*p* < 0.05). **(C)** mRNA expression of FASN was markedly downregulated by miR-144-5p treatment (*p* < 0.05) in HFD-induced obese mice. **(D)** mRNA expression of CPT1 was upregulated by miR-144-5p treatment in obese mice. **(E)** mRNA expression of ACOX1 was upregulated by miR-144-5p treatment in obese mice. Data shown are mean ± SD. *p < 0.05, **p < 0.01, relative to control group, #*p* < 0.05, ##*p* < 0.01, relative to model group.

The results indicated that miR-144-5p intervention significantly enhanced CPT1 activity (*p* < 0.01) and increased the hepatic FAO rate in the liver of obese mice (*p* < 0.05; Figures 10A,B). The qRT-PCR results demonstrated that the mRNA expression of FASN, which is involved in fatty acid uptake, was markedly downregulated by miR-144-5p treatment (*p* < 0.05; Figure 10C), indicating inhibition of fatty acid synthesis in HFD-induced obese mice. Conversely, the expression of CPT1, the rate-limiting enzyme in fatty acid *β*-oxidation, along with ACOX1, which is involved in the peroxisomal β-oxidation of very long-chain fatty acids, were upregulated by miR-144-5p treatment in obese mice (Figures 10D,E), suggesting enhanced mitochondrial and peroxisomal β-oxidation, respectively.

Collectively, these results suggest that miR-144-5p alleviates obesity-associated lipid accumulation by regulating key metabolic enzymes that promote fatty acid oxidation in diet-induced obese mice, potentially through the regulation of key metabolic enzymes promoting fatty acid oxidation.

## 4 Discussion

The Bactrian camel (*Camelus bactrianus*) illustrates remarkable adaptations for surviving extreme environments by efficiently converting stored fat into energy and water, highlighting the critical role of lipid metabolism regulation (17). Obesity, characterized by metabolic dysfunction and excessive fat accumulation, shares key pathways with these adaptive mechanisms. Recent studies have revealed the role of exosomes in facilitating interactions between adipose tissue and other organs (18). Building on these insights, our study reveals that miR-144-5p is pivotal in modulating obesity-related metabolic dysfunctions. We found that administration of miR-144-5p to a HFD-induced obese mouse resulted in reduced body weight, improved lipid profiles and fatty acid oxidation, confirming its therapeutic potential and aligning with prior findings that underscore the influence of miRNAs on metabolic pathways (5, 19). These results bridge evolutionary biology with clinical applications, offering insights for developing obesity treatment strategies. Specifically, the exosomal miRNAs involved in fat regulation provide novel molecular insights into metabolic disorders associated with obesity.

The expression levels of miRNAs in obese individuals suggest their potential as biomarkers for assessing metabolic status (20). Building upon previous research, which identified 40 differentially expressed miRNAs in plasma exosomes from two groups of Bactrian camels with varying body types (2), our study highlights a significant differential expression of miR-144-5p, pointing to its crucial role in lipid metabolism regulation (21, 22). Consistently, miR-144-5p exhibited high conservation across multiple species, emphasizing its essential role in growth and development (23). Through bioinformatics analysis, target gene prediction, and validation experiments, we demonstrated that miR-144-5p targets the PGC-1α, a key regulator of browning, thermogenesis, and energy metabolism in brown and beige adipocytes (24). This was confirmed using RNA pull-down and luciferase reporter assays. Furthermore, KEGG analysis through bioinformatics tool suggested that it was closely related to lipid metabolism and AMPK pathways. Despite the typical role of PGC-1α in promoting mitochondrial biogenesis and fatty acid oxidation (25), its regulation by miR-144-5p provides a different perspective on lipid metabolism.

Our *in vivo* experiments revealed that miR-144-5p administration ameliorated HFD-induced weight gain and hepatic steatosis, suggesting potential hepatoprotective effects. Additionally, it attenuates obesity-associated inflammatory responses. These findings highlight the dual role of miR-144-5p in modulating lipid metabolism and inflammation, consistent with its involvement in the pathogenesis of non-alcoholic fatty liver disease (NAFLD) and other liver disorders (26). However, its tissue-specific functionality is evident, as it contrasts with some reported pathological roles in diabetes (27), indicating a more complex role of miR-144-5p in different metabolic contexts.

Intriguingly, while miR-144-5p negatively regulates PGC-1α, its intervention paradoxically improved fat metabolism and reduced body weight in obese mice. Our results showed inhibition of PGC-1α expression yet upregulation of CPT1 and ACOX1, along with downregulation of FASN, suggesting a potential therapeutic effect targeting both lipogenesis and *β*-oxidation. These data led us to several hypotheses, potentially explaining why intervention of miR-144-5p ameliorated lipid metabolism despite the suppression of PGC-1α expression: First, miR-144-5p may target other factors within the metabolic network, compensating for the suppression of PGC-1α. Second, even with suppressed expression of PGC-1α, an incomplete activation of the pathway could result in observed metabolic benefits. Third, the biological system’s compensatory mechanisms might activate alternative pathways, such as AMPK, enhancing energy metabolism despite PGC-1α suppression. AMPK pathway has been proved by several reports to exert regulatory impacts on FAO metabolism (28, 29). Lastly, miR-144-5p may has differential impacts across various tissues, as different cell types might exhibit distinct regulatory responses, leading to overall metabolic improvements.

Although miR-144-5p may partially impair mitochondrial function and fatty acid oxidation by suppressing PGC-1α, it can still ameliorate obesity-associated lipid accumulation overall through mechanisms such as activating AMPK, suppressing inflammatory or lipogenic pathways. This multi-target and multi-pathway regulatory mode highlights the complexity and potential therapeutic value of miRNAs in metabolic diseases. Future studies should integrate tissue-specific gene editing and dynamic metabolomics to further elucidate its molecular network.

Although this study provides significant insights into the role of miR-144-5p in lipid metabolism, several potential limitations should be acknowledged. First, the exclusive use of male mice limits the generalizability of the findings across sexes. Sex-dependent differences in metabolic outcomes, often influenced by sex hormones, necessitate investigations in female subjects to ensure comprehensive understanding (30). Second, the regulatory network analyzed is incomplete. This study did not comprehensively explore the upstream and downstream molecular mechanisms involved in the regulation by miR-144-5p.

Despite these limitations, our study presents several strengths. We used a well-established mouse model of obesity, providing a reliable platform for studying the impact of miRNA regulation on metabolic parameters. Rigorous monitoring of health indicators such as body weight and blood lipid levels ensured precise characterization. Furthermore, this study is the first to document the downregulation of PGC-1α in obese mice following miR-144-5p intervention, exploring the miR-144-5p/PGC-1α axis in the context of obesity. Notably, our findings may extend beyond murine models to suggest evolutionary conserved mechanisms. The observed upregulation of miR-144-5p in normal-weight Bactrian camels compared to lean camels could reflect an adaptive “lipid-storing” phenotype. Crucially, miR-144-5p likely appears to function as a context-dependent metabolic modulator: under physiological conditions, it facilitates lipid storage while preserving systemic homeostasis; however, during pathological obesity, it may paradoxically enhance lipolytic capacity to counteract excessive adipogenesis. This bidirectional regulatory mechanism suggests miR-144-5p’s critical role in maintaining metabolic flexibility across physiological and pathophysiological states.

In conclusion, our study provides new insights into the influence of miR-144-5p on lipid metabolism in HFD-induced obese mice, demonstrating that miR-144-5p intervention improves lipid metabolism in obese male mice. To further understand these results, several areas deserve deeper exploration: Further examination of different metabolic pathways might identify additional genes targeted by miR-144-5p; Observing other biomarkers, including hormone levels (e.g., insulin, adrenaline), fatty acid composition, and glucose metabolism indices in the intervention group, could unravel detailed metabolic processes; Long-term studies on miR-144-5p intervention could determine the sustainability of metabolic changes or adaptive adjustments over time. These further analyses will enhance our understanding of the complex role of miR-144-5p in obesity and lipid metabolism, potentially unveiling underlying mechanisms.

## 5 Conclusion

This study highlights the pivotal role of Bactrian camel plasma exosomal miR-144-5p in regulating lipid metabolism and its interaction with inflammation in the context of obesity. Through a well-established mouse model of HFD-induced obesity, we demonstrated that miR-144-5p administration significantly reduces fat accumulation, improves lipid profiles, and mitigates the inflammatory responses, providing promising therapeutic insights for addressing metabolic dysfunctions associated with obesity.

Furthermore, our findings suggest that the therapeutic targeting of miR-144-5p may offer a promising approach for the prevention and treatment of obesity and its complications. However, future research should focus on comprehensively investigating additional targeted genes, analyzing various metabolic pathways, and assessing other pertinent biomarkers, including hormonal and metabolic indices, to unravel the detailed mechanisms through which miR-144-5p influences obesity-related metabolic disorders. Overall, our research contributes to the growing understanding of the complex relationship between microRNAs and metabolic regulation, highlighting miR-144-5p as a potential biomarker and therapeutic target in obesity management.

## Data Availability

The datasets presented in this study can be found in online repositories. The names of the repository/repositories and accession number(s) can be found at: https://www.ncbi.nlm.nih.gov/geo/, GSE197066.

## Footnotes

^1^ www.mirbase.org

^2^ http://blast.ncbi.nlm.nih.gov/Blast.cgi

^3^ https://www.genome.jp/tools-bin/clustalw

^4^ http://www.targetscan.org/

^5^ http://mirwalk.umm.uni-heidelberg.de/

^6^ https://david.ncifcrf.gov/

^7^ https://www.kegg.jp/

^8^ https://www.bioinformatics.com.cn
