# Supplementary material for: Role of miR-144-5p in modulating lipid metabolism and potentially alleviating obesity via the PGC-1α/AMPK pathway
Source: Front Vet Sci. 2025 Aug 14;12:1477593. doi: 10.3389/fvets.2025.1477593 (PMC12392996; doi:10.3389/fvets.2025.1477593)
Supplement: Supplementary file 1 [file Table_1.docx]

Supplementary Material

## Supplementary Table


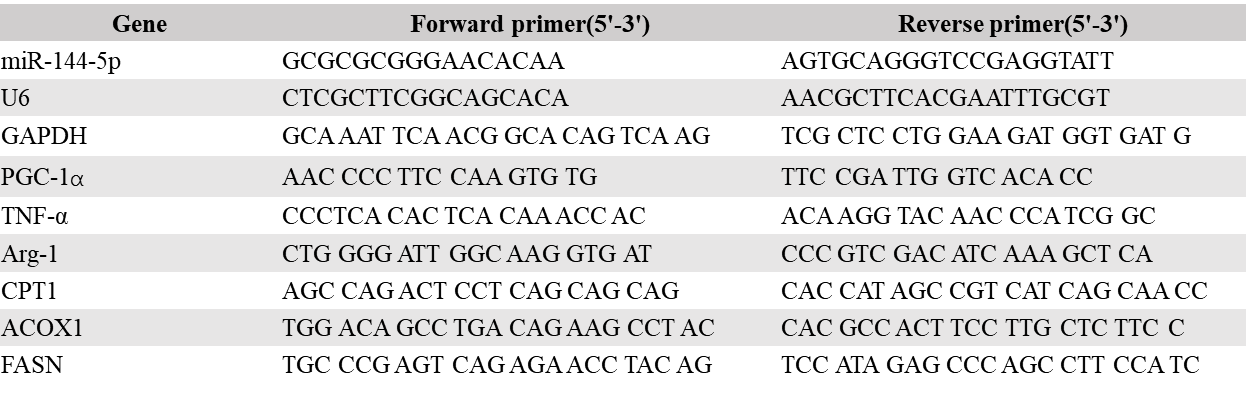


**Supplementary Table 1.** Primer sequences used for real-time quantitative PCR.

miR-144-5p, microRNA 144-5p; U6, U6 small nuclear RNA; GAPDH, glyceraldehyde-3-phosphate dehydrogenase; PGC-1α, peroxisomal proliferatoractivated receptor g coactivator-1α; TNF, tumor necrosis factor; Arg-1, arginase 1; CPT1, carnitine palmitoyltransferase-1α; ACOX1, acyl-CoA oxidase 1; FASN, fatty acid synthase.
